# Influence of linguistic properties and hearing impairment on visual speech perception skills in the German language

**DOI:** 10.1371/journal.pone.0275585

**Published:** 2022-09-30

**Authors:** Nina Suess, Anne Hauswald, Verena Zehentner, Jessica Depireux, Gudrun Herzog, Sebastian Rösch, Nathan Weisz

**Affiliations:** 1 Centre for Cognitive Neuroscience and Department of Psychology, University of Salzburg, Salzburg, Austria; 2 Salzburger Landeskliniken, Salzburg, Austria; 3 Department of Otorhinolaryngology, Head and Neck Surgery, Paracelsus Medical University Salzburg, University Hospital Salzburg, Salzburg, Austria; Max-Planck-Institut fur Kognitions- und Neurowissenschaften, GERMANY

## Abstract

Visual input is crucial for understanding speech under noisy conditions, but there are hardly any tools to assess the individual ability to lipread. With this study, we wanted to (1) investigate how linguistic characteristics of language on the one hand and hearing impairment on the other hand have an impact on lipreading abilities and (2) provide a tool to assess lipreading abilities for German speakers. 170 participants (22 prelingually deaf) completed the online assessment, which consisted of a subjective hearing impairment scale and silent videos in which different item categories (numbers, words, and sentences) were spoken. The task for our participants was to recognize the spoken stimuli just by visual inspection. We used different versions of one test and investigated the impact of item categories, word frequency in the spoken language, articulation, sentence frequency in the spoken language, sentence length, and differences between speakers on the recognition score. We found an effect of item categories, articulation, sentence frequency, and sentence length on the recognition score. With respect to hearing impairment we found that higher subjective hearing impairment is associated with higher test score. We did not find any evidence that prelingually deaf individuals show enhanced lipreading skills over people with postlingual acquired hearing impairment. However, we see an interaction with education only in the prelingual deaf, but not in the population with postlingual acquired hearing loss. This points to the fact that there are different factors contributing to enhanced lipreading abilities depending on the onset of hearing impairment (prelingual vs. postlingual). Overall, lipreading skills vary strongly in the general population independent of hearing impairment. Based on our findings we constructed a new and efficient lipreading assessment tool (SaLT) that can be used to test behavioral lipreading abilities in the German speaking population.

## 1 Introduction

Evidence that visual cues help to understand speech under noisy conditions has existed for a long time [1] and since the discovery of the McGurk effect [2], researchers became aware that there might be an important contribution from the visual system to speech perception. Indeed, neuroimaging studies about integration of audiovisual speech cues (see [3] for a review) provide evidence for enhanced comprehension of speech under noisy conditions when presented with the speaker’s face [4]. Also, the acoustic speech envelope and lip movements are highly correlated, providing evidence that they carry common information for the listener [5, 6]. Although this correlation is strong, it is not perfect, thus raising the question of how visual cues contribute to speech understanding. Interestingly, observing the speaker’s face without auditory input showed processing of the unheard speech envelope accompanying lip movements [7]. This implies that the brain can infer acoustic features from the visual input, known as visuo-phonological transformation [7]. Therefore, the integration of cues coming from different modalities would seem important to understand speech under adverse conditions. Carrying those notions forward to the linguistic perspective also adds evidence that there seems to be a difference between visual and auditory speech: While clear auditory speech includes a number of clearly distinguishable phonetic units, the same does not hold true for the visual companion. From the visual perspective, it is difficult to differentiate e.g. voiced and unvoiced consonants (e.g. /b/ and /p/, /g/ and /k/) [8] or also the consonants /b/ and /m/. But while it is difficult, it is not entirely impossible as studies provide evidence that both on a behavioral and neural level those perceptually almost identical consonant-vowel combinations can be differentiated [9, 10].

Nevertheless, those phonemes or consonant-vowel combinations that are perceptually similar in terms of visual perception are grouped in units called visemes [11]. There are far less visemes than phonemes in languages in general (for English see [12]), making it harder to recognize speech by visual cues alone. Consequently, if phonetic information is reduced in visual speech but skilled people still understand speech alone by visual cues (e.g. [13] report up to 70% correct), this raises the question: Which other factors contribute to successful lipreading? Interestingly, people show in general low accuracy for lipreading of naturalistic stimuli [14] when the context is missing. This may be connected to the fact that without lexical restriction of possible phonological information, visemes are delivering ambiguous information, hence making it harder to infer the correct words from visual input alone. Therefore, one influential factor could be the frequency of occurrence of the words in the spoken language. Few studies investigated this factor and found that high-frequency words (words that are used often in the spoken language) are recognized more often than low-frequency words [15]. And as already mentioned, some phonemes have similar visual articulatory characteristics, and therefore the place of articulation could also be crucial for successful lipreading [16]. Compellingly, reading and language skills seem to correlate to some extent with lipreading abilities [17, 18], signifying possible interactions with education as well. Another consideration that is mentioned to contribute to altered lipreading abilities may be the extent of diminished hearing abilities. This causes hearing impaired people to rely more on visual cues for speech processing and to show superior lipreading abilities [19–22]. Notably, most of these studies worked with early-onset (and completely) deaf individuals, omitting the group of people with postlingual acquired hearing loss. Since hearing impairment and aging go hand in hand, those two factors could also be a crucial prerequisite for enhanced lipreading skills as a compensatory mechanism for preserved speech understanding. Contrary to postlingual acquired hearing loss stands the group of prelingually deaf individuals who do not experience gradual hearing loss with age, therefore missing the process of “perceptual compensation” [23]. This absence may also have an impact on how lipreading skills are evolving over time in prelingually deaf individuals, something that may explain that early studies support a controversial point of view about enhanced lipreading skills in prelingual deaf people [24, 25].

A closer inspection of the mentioned studies revealed the use of different approaches for measuring lipreading abilities since there has not been a widely used assessment tool. Although there was a lot of effort taken to construct English lipreading tests (e.g. Utley and colleagues [26]) using word, sentence, and story recognition with high reliability and validity scores, or Bannister & Britten [27], building on the test from Utley and colleagues to develop the EASL), there have hardly been any for the German language. Especially in recent years, the behavioral assessment of visual speech perception has not received extensive attention (regardless of the language studied). Therefore, we aimed to construct a tool for measuring lipreading abilities using everyday and easy-to-understand German words and sentences. Our goal in this study was (1) to identify factors that contribute to better understanding of visual speech (both intrapersonal and from a linguistic perspective) and (2) to provide a time-effective tool that is successful in distinguishing lipreading abilities between subjects. We used stimuli from already established acoustic speech understanding assessments which are widely used in Austrian ENT-clinics. We presented participants silent videos from stimuli of those speech understanding assessments and investigated how people could extract linguistic information from silent lip movements. To measure hearing impairment, we used an already established questionnaire (APHAB, [28]) which is usually used for assessing hearing aid benefit, but includes mostly everyday-life questions, which is appropriate for our purpose. Based on this hearing impairment assessment, we also tried to evaluate the distinctions between different subjective hearing impairment levels and we wanted to identify the factors that influence visual speech perception abilities. We hypothesize that the viseme category has an influence in both sentences and word recognition, and that also the use-frequency of the words in spoken language has an influence. We also investigated differences between different versions of our assessment. Moreover, we hypothesize that hearing impairment has an influence on the total test score and the sentence score alone and that this relationship could be moderated by education or age. After testing our hypotheses, we evaluated the data by fitting a Rasch model and an exploratory factor analysis to reduce the items while still being able to measure lipreading skills. The identified items are now used in the new test called SaLT (Salzburg Lipreading Test) to offer a time effective tool for examining lipreading abilities in the German language.

## 2 Materials and methods

### 2.1 Participants

The participants were recruited for the experiment via social media and on the university campus. 170 participants (135 normal hearing; 120 females; mean age: 34.5 years; SD: 14.07 years, range: 18–71) completed the whole test. Hearing impaired participants (N = 13, APHAB score > = 34 and < = 90) were mainly acquired via contact with our ENT specialist, prelingually deaf participants (N = 22) were acquired via their general practitioner at the hospital. The general practitioner only chose individuals who did not wear hearing aids or cochlear implants, thus not having received auditory input throughout their lifetime to make sure that their experiences were comparable within their group. All participants were asked to state whether they have a diagnosed hearing impairment. If there was, they were also requested to report on how long the impairment had been present and whether it was prelingually or postlingually acquired. Because of technical difficulties, the answers for the number-items were not recorded in 4 cases. We decided to keep them for the analysis on the words and sentences part, but excluded them for the analysis on the total score (N = 166). Psychology students received credits for their participation. All participants provided written informed consent and were able to abort the experiment at any time by closing the window of their browser. The experimental procedure was approved by the Ethics Committee of the University of Salzburg (GZ 5/2019).

### 2.2 Stimuli

Four different speakers (2 male, 2 female) recorded videos of all stimuli that were chosen according to the later described item categories. The videos were taken in front of a light gray background with 50 fps. The editing software that was used was DaVinci Resolve 15.3.1. The videos were edited to such a degree that the mouth of the speaker was closed when the video started and closed again at the end of each video. There were 4 sets of each video type (numbers, words, sentences). Four different versions of stimulus sets were created, each with one female and one male speaker. The number of videos of female and male speakers were also balanced for each set. Each item was presented only once, and all participants were presented with the same items. The order of the items and the speaker who presented the items were pseudorandomized. Items were taken from pre-established audio speech understanding tests since they use words and sentences which are used in everyday-life and should therefore be familiar to participants. This was done to avoid any misunderstandings based on linguistic knowledge gaps (e.g. not being familiar with certain words). The next sections briefly describe the speech understanding tests from which the items were drawn.

#### 2.2.1 “Freiburger Sprachtest”–„Freiburger speech test”

The Freiburger speech test [29] is a German language test for acoustic speech understanding. It includes 100 polysyllabic numbers and 400 monosyllabic every-day substantives of which 18 numbers and 48 words were used.

#### 2.2.2 “HSM Satztest”–“HSM sentence test”

The HSM sentence test by Hochmair-Desoyer et al [30] is a German language test for acoustic speech understanding. It includes 600 every-day sentences of which 36 sentences were used.

#### 2.2.3 Datenbank für gesprochenes Deutsch (DGD)–Database for spoken German

To acquire the frequency of single words in the German spoken language, we looked for a database that records information on spoken German. The “Datenbank für gesprochenes Deutsch–DGD”(English: „Database for spoken German“) is a corpus management system and part of the “Programmbereich Mündliche Korpora des Instituts für Deutsche Sprache”(English: „Program for oral corpora of the institute for German language“) [31] For this assessment, we used the version 2.12 (release date: May 2019). It consists of data from different areas of social life, such as work, leisure time, education, etc., that is transcribed from audio data. The total number of data in version 2.12 amounts to 306 different conversations with 250.5 hours of audio recordings and 2.43 million transcribed tokens. The frequency of all words used in the lipreading test (either as stand-alone words, or as words in a sentence) were extracted. This was then used to later calculate the Zipf score to obtain the final word frequency independent of the size of the word corpus (section “Frequency—Zipf score”).

### 2.3 Item selection

#### 2.3.1 General selection

Specific characteristics were defined to classify the difficulty of the presented items: Word- frequency, sentence-frequency, articulation, and sentence length. To be able to compare high-, medium and low-frequency words, there was an equal number of bilabial and non-bilabial words in each of those three groups to make sure that any effect was related to the frequency but not to articulation. For words, that resulted in 6 categories: for each of the three frequency-categories, there were the 8 bilabial and 8 non-bilabial words, so 48 words in the whole test. The frequency categories differed significantly from each other for the bilabial words (F(1, 22) = 154.60, *p <* .*001*) and also for the non-bilabial words (F(1, 22) = 1105.00, *p <* .*001*).

We divided the sentences in 3 length categories, resulting in 12 sentences each, with 4 in each frequency category, providing a total number of 36 sentences. The length categories differed significantly from each other (F(2,33) = 161.20, *p >* .*001*), and also the frequency categories differed significantly from each other (F(2, 33) = 85.05, *p <* .*001*).

The chosen items can be found in the S1–S4 Tables. Detailed selection criteria are following in the next respective sections.

#### 2.3.2 Frequency—Zipf score

All audio files of the “Freiburger Sprachtest” and the HSM were transcribed and every word was then assigned with a score that displayed the frequency of appearance in the DGD corpus. For this purpose, the Zipf score was used [32]. This score is a measure for word-frequency based on a logarithmic scale with values between 1 and 7 which can be used independently of the size of the word corpus it is used upon. The Zipf score is calculated using the following formula:

$$Zipf=\left(\frac{FrequencyCount+1}{\frac{tokens}{1,000,000}+\frac{types}{1,000,000}}\right)+3$$

Type, in this context, refers to the amount of different words in a corpus. For example, the sentence “What this is, is this.” contains 5 tokens, but only 3 types (“what”, “this”, and “is”). We introduced 3 frequency categories (high-frequency, medium-frequency and low-frequency words) to be able to distinguish between frequency-categories and chose the words accordingly. For sentence items, we took the Zipf score per word from the DGD and calculated an average Zipf score to obtain high-frequency, medium-frequency and low-frequency sentences. For calculating statistics, the categories were abandoned again and the exact Zipf score was used.

#### 2.3.3 Articulation

For words, two articulation categories were created: bilabial and non-bilabial. Words that start with /b/, /p/, or /m/ were defined as bilabial. All other words were defined as non-bilabial. For sentences, this differentiation was not made.

#### 2.3.4 Sentence length

For sentences, three different categories of length were created. The shortest sentence had 3 words, the longest 9. Short sentences had 3 or 4 words, medium sentences had 5 or 6 words and long sentences had 7, 8 or 9 words. For calculating statistics, the categories were abandoned again and the exact sentence length score was used.

### 2.4 Procedure

#### 2.4.1 General procedure

The study was conducted online in LimeSurvey. Instructions were given in written form before the participants started the survey on their own. They were instructed to conduct the survey in a quiet environment and to use a PC with a big screen to avoid difficulties due to small screens (e.g. on a smartphone). Furthermore, they were told to not hurry when completing the test, since this could lead to errors in playing the video because of internet connection issues. In the beginning we asked to give demographic information. Then they were asked on a scale from 1 to 5 how highly they rated their lipreading ability. They were asked the same question again after completing the study.

#### 2.4.2 APHAB

To document if there was a subjective hearing impairment, they also filled out the APHAB (Abbreviated Profile of Hearing Aid Benefit, [28]). As we were not able to test an objective measure of hearing impairment, we decided to use this scale since it includes questions where participants rate 24 everyday situations where one might have hearing problems (for example: “It is hard for me to understand dialogs at the movies or the theater.”) on a scale of 1 to 7—from “always” to “never”. The 7 levels of the scale are represented by percentages from “always” representing “99%” and “never” representing “1%”. The higher the percentage over all items is, the stronger the subjective hearing impairment. Prelingual deaf people that participated were instructed to answer only the first item with “never” and skip the rest of the questions so that they could be identified. They were then assigned the highest possible score in the APHAB (99) to reflect the complete absence of hearing.

#### 2.4.3 Lipreading task

After the participants completed the APHAB, they were presented randomly with one of the four versions of the test. As mentioned in the section “Stimuli”, one version of the test consisted of 1 male and 1 female speaker. The participants were told that there will be three item categories: First the numbers, then the words and then the sentences. They could decide autonomously when the video of an item should start by pressing a “Play”-button in the middle of the screen. Each video could be viewed twice. The videos were not presented more often to imitate the real-life trait of lipreading accurately. We decided against only one presentation as a safeguard against attentional lapses and to make the experience less discouraging for people with low lipreading skills. They were asked to write down exactly the words they could understand from the videos in a response box below the video and they were also encouraged to give partial responses. They could also delete and type the responses again without a time limit. It was also possible for the participants to not give an answer. There was no feedback on the performance.

#### 2.4.4 Speaker intelligibility

After the completion of all items, the participants were presented with pictures of all speakers. They were asked to rate on a scale of 1 to 4 how well they understood the speakers that they saw in the videos (so to just rate 2 of the presented 4 speakers).

### 2.5 Data analysis

#### 2.5.1 Evaluation of test results

The test results were evaluated by one of three raters, **resulting in one score per item and participant**. The rating was done manually instead of automatically for two reasons: when a participant answered correctly but a spelling mistake was included in their answer, the answer was to be evaluated as correct. Parts of words that were correct were also taken into account, as described further in this section.

*2*.*5*.*1*.*1 Numbers*. For numbers, answers could be rated either 0%, 50% or 100%. An answer was rated 0% if it was incorrect as a whole or if no answer was given, 50% if one part of the two-part number was answered correctly and 100% if the number given as an answer was exactly the same as the number pronounced by the speaker. For example, if the correct answer was 65 (“Fünf-und-Sechzig”) and the response was 23 (“Drei- und-Zwanzig”), the rating was 0%. If the response was, for example, 35 (“Fünf-und- Dreißig”), 63 (“Drei-und-Sechzig”), or even 50 („Fünf-zig“) the rating was 50%. Note that an answer like 53 (“Drei-und-Fünfzig”) was rated 0%, because even though “Fünf” is a correct part of the word, it is in the wrong position. Then the mean of all number scores was calculated to form the overall percentage of correct answers for each participant. Henceforth, this averaged percentage will be addressed as the test score for numbers.

*2*.*5*.*1*.*2 Words*. Words could be rated either 0%, 50% or 100%. If the whole word was correct or just differed by a spelling mistake and the intention for the right word was clear, the test score was 100%. If either the first or last viseme was identified correctly, the answer was rated 50%. For example, if the correct answer was “Baum”, answers like “Bauch” or “Flaum” were rated 50% (because in “Bauch”, the first part of the word was identified correctly, while in “Flaum”, the second part of the word was identified correctly). As the phonemes “Ma” and “Ba” cannot be distinguished just by visual inspection, they were also rated correct when confused (e.g. if the word was “Mann” and the answer was “Band”, the first phoneme was recognized and therefore the word was rated 50% correct). Then the mean of all word scores was calculated to form the overall percentage of correct answers for each participant. This averaged percentage will be addressed as the test score for words. The rating of answers as either fully- or half- correct is comparable to the scoring in the speech-in-noise test by Killion and colleagues [33]. In very rare cases, an answer was rated either 25% correct or 75% correct. This happened when e.g. for “Baum”, the answer was “B”, indicating that the beginning of the word was understood, indicating that they correctly lipread the letter “B”. A rating of 75% was given when the whole word except for one letter was correct, e.g. for the word “Molch”, the response was “Mölch”. Although the word was not completely understood, it was rated 75% because it was identified almost completely. To avoid uncertainties when using our assessment tool, we uploaded a list of particular scoring examples which will be available on the OSF-page (see https://osf.io/sgj4n/).

*2*.*5*.*1*.*3 Sentences*. For sentences, each word in a sentence was rated either correct (100%) or incorrect (0%). An exception were double-words, which are common in the German language. If one of the words of a double-word was in the answer, it was rated “half-correct” (50%). For example, if in the correct sentence, the word “Bauchtanz” (Bauch + Tanz) was included, if the participant’s answer included either “Bauch” or “Tanz”, that word was rated 50%. We then averaged the percentages of all words in a sentence, which could span from 0% to 100%. Then the mean of all sentence scores was calculated to form the overall percentage of correct answers for each participant. The averaged percentage will be addressed as the test score for sentences.

#### 2.5.2 Statistical evaluation

*2*.*5*.*2*.*1 Item recognition*. We first calculated the mean recognition score per number, per word and per sentence independent of participants. This resulted in 18 mean scores for numbers, 48 mean scores for words and 36 mean scores for sentences. We then calculated a Friedman ANOVA to assess possible differences between item categories (numbers, words, and sentences) and further investigated those effects using paired Wilcoxon signed-rank tests with Bonferroni correction using the *stats* package in [34] This comparison has been done in order to investigate if a different number of the possible solutions has an impact on the recognition score.

We then investigated all other possible influential factors on the item recognition score by using the recognition scores per individual word and sentence dependent on participants.

To investigate if the articulation category or the Zipf score had an impact on the recognition score (in %) of words, we calculated a general linear mixed model with the package *lme4* [35] in R [34]. The fixed effects were defined as *Zipf score* (continuous variable) and *Articulation* (categorical with two levels), including an interaction term between those predictors. To account for the dependency between observations over participants, we modeled responses by the same person with varying intercepts. We furthermore centered the predictor *Zipf score* to avoid multicollinearity and make interpretation easier [36].

To investigate if the sentence length or the Zipf score had an impact on the recognition score (in %) of sentences, we calculated a general linear mixed model with the package *lme4* [35] in R [34]. The fixed effects were defined as *Zipf score* (continuous variable) and *Sentence length* (continuous variable), including an interaction term between those covariates. To again account for the dependency between observations over participants, we modeled responses by the same person with varying intercepts. Here, we centered both predictors.

Further to this, we tested possible differences between the 4 different versions of the test using the Kruskal-Wallis test.

*2*.*5*.*2*.*2 Influence of hearing impairment*. To assess if differences in subjective hearing impairment have an influence on the test score, we calculated a linear regression analysis where hearing impairment (APHAB score) was the predictor and the total test score the dependent variable. We also tried to predict the total test score for the prelingually deaf (N = 22) people from the sample with people with postlingual acquired hearing loss using the function *predict* from the *car* package [37] To see if the relationship between hearing impairment and test score was influenced by other factors, we calculated moderator analyses with the variables education and age once for the group without prelingually deaf participants and once for the total sample. Furthermore, we calculated the moderator analyzes again just for the sentence score to investigate influences of our moderators just for naturalistic stimuli with a certain grammatical structure. For the moderator analyses, we centered both the independent variable and the moderator variable [36].

*2*.*5*.*2*.*3 New version (SaLT—Salzburg Lipreading Test)*. To reduce the number of previously utilized items, we used the item response theory (IRT) and estimated a dichotomous Rasch model. The Rasch model uses “false” and “correct” as two categories where the respective item is mapped onto and shows the different probabilities for solving the item dependent on the latent variable (trait) that should be measured (in our case lipreading abilities). We decided to count all answers that were between 100% and 51% correct as “correct” and all answers between 50% and 0% as “false” and then fitted a Rasch model (RM) separately for numbers and words with all the items that were included in the first version of our assessment using the *eRm* package [38]. We first used the function *stepwiseIt* to eliminate items based on the item fit to check for the independence of the item parameters from the persons tested in our sample by calculating the person ability parameters. If this function either eliminated too little or no items, we continued with calculating the Andersen LR-test, which also compares the response patterns of subgroups and checks if all items have the same selectivity (“Trennschärfe”) and therefore can display the characteristics (“Merkmalsausprägung”) of our latent trait over the whole testing population equally. A p-value under .05 indicates that the assumption of objective specificity is violated and therefore the parameter estimates are not equal across subgroups. When an item was excluded by the package because of inappropriate response patterns within subgroups, we excluded it before fitting a new model again with fewer items. We fitted a last RM to check again with the item fit and the Andersen LR-test if our remaining items are still able to measure our latent variable.

For the reduction of sentences, we used a different approach. Since the test score for sentences can range from 0 to 100% and can result in different scores due to the averaging over single words, we decided to use an exploratory factor analysis (EFA), which is optimal for the reduction of items that have a continuous scale. This analysis was done using the *psych* package [39]. All 36 sentence items were included into an exploratory factor analysis with the minimum residual factoring method and orthogonal rotation (varimax). We predefined the number of factors to be 1 because we assumed that relevant items are just loading on the factor “lipreading abilities”.

Finally, analyses of internal consistency with Cronbach’s alpha were conducted for numbers, words and sentences separately to measure internal reliability of different item categories. All analyses in this section were conducted using R [34].

## 3 Results

### 3.1 Impact of linguistic factors

#### 3.1.1 Item categories

**Fig 1:**
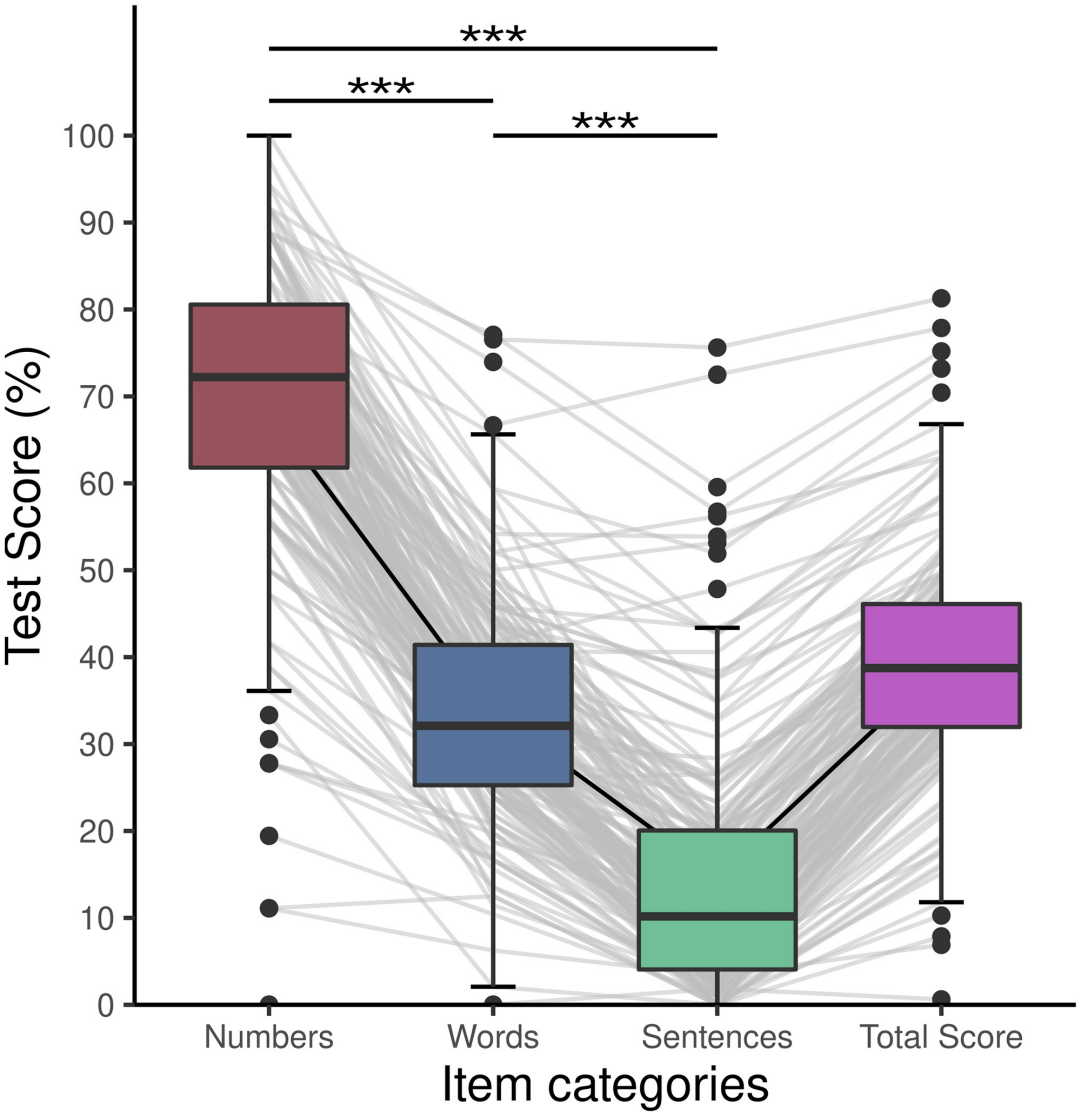
Differences between item categories and total score. The proportion of isolated numbers correct was higher than the proportion of isolated words correct or words correct in sentences. Also the proportion of isolated words correct was higher than the proportion of words correct in sentences. Asterisks depict significant values p < .001. Gray lines depict individual subject values.

Whereas the recognition rate for the numbers were high (N = 166, M = 68.43%, SD = 17.80%, range = 0–100%), lipreading abilities for complex stimuli were low in general (N = 170, words: M = 33.62%, SD = 13.18%, range = 0–77.08%; sentences: M = 14.75%, SD = 14.90%, range = 0–75.61%). Participants who completed the whole test (N = 166) had on average a total test score of 38.93% (SD = 13.42%, range = 0–81.28%). In order to compare the item categories statistically, we calculated a Friedman’s test. Our results show significant differences between the item categories (Χ^2^_F_ = 311.93, *p <* .*001*). Post-hoc Wilcoxon signed-rank tests with Bonferroni-correction revealed significant differences between all categories (*p <* .*001* for all contrasts, Fig 1). A similar analysis using a general linear mixed model can be found in the S5 Table.

#### 3.1.2 Words

**Fig 2:**
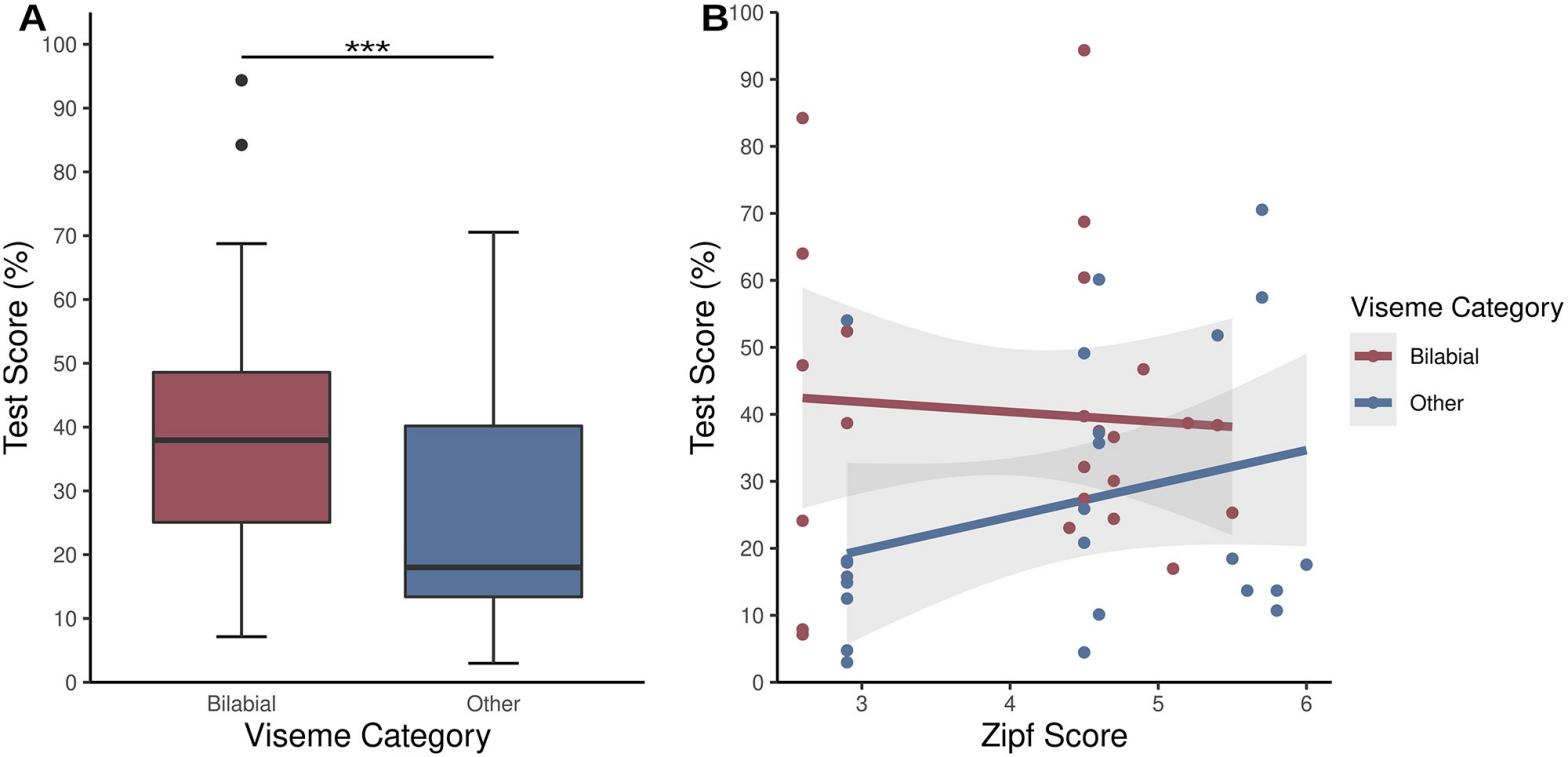
Impact of linguistic factors on the recognition score of words. A) Impact of viseme category on word recognition. Bilabial words (red) contribute more to the recognition score than other words (blue, p < .001). B) Impact of Zipf score on word recognition of each word dependent on the viseme category. The Zipf score had a significant impact on the word recognition (p < .001), but just in the category where no bilabial cue was present, showing a significant interaction between Zipf score and viseme category (p < .001).

We then tested whether the main factors “articulation of the monosyllabic words” and “Zipf score” had an impact on the word recognition score and if those main factors show an interaction. The mean score for bilabial words was 40.26% (SD = 21.83%, range = 7.14–94.35%) and for non-bilabial words 26.59% (SD = 20.06%, range = 2.98–70.54%). We found a significant main effect of the articulation category (*β* = -.462, SE = .004, *p <* .*001*), meaning that the articulation category could predict the recognition score of words (Fig 2A) and we also found a significant main effect of the Zipf score (*β* = -.033, SE = .002, *p <* .*001*), meaning that the word frequency could predict the recognition score of words. We also found a significant interaction effect between the viseme category and the Zipf score (*β* = .22, SE = .003, *p <* .*001*), showing that only in the absence of a bilabial cue, the Zipf score had an impact on the recognition score (Fig 2B). The table with the fixed effects can be found in the S6 Table.

#### 3.1.3 Sentences

**Fig 3:**
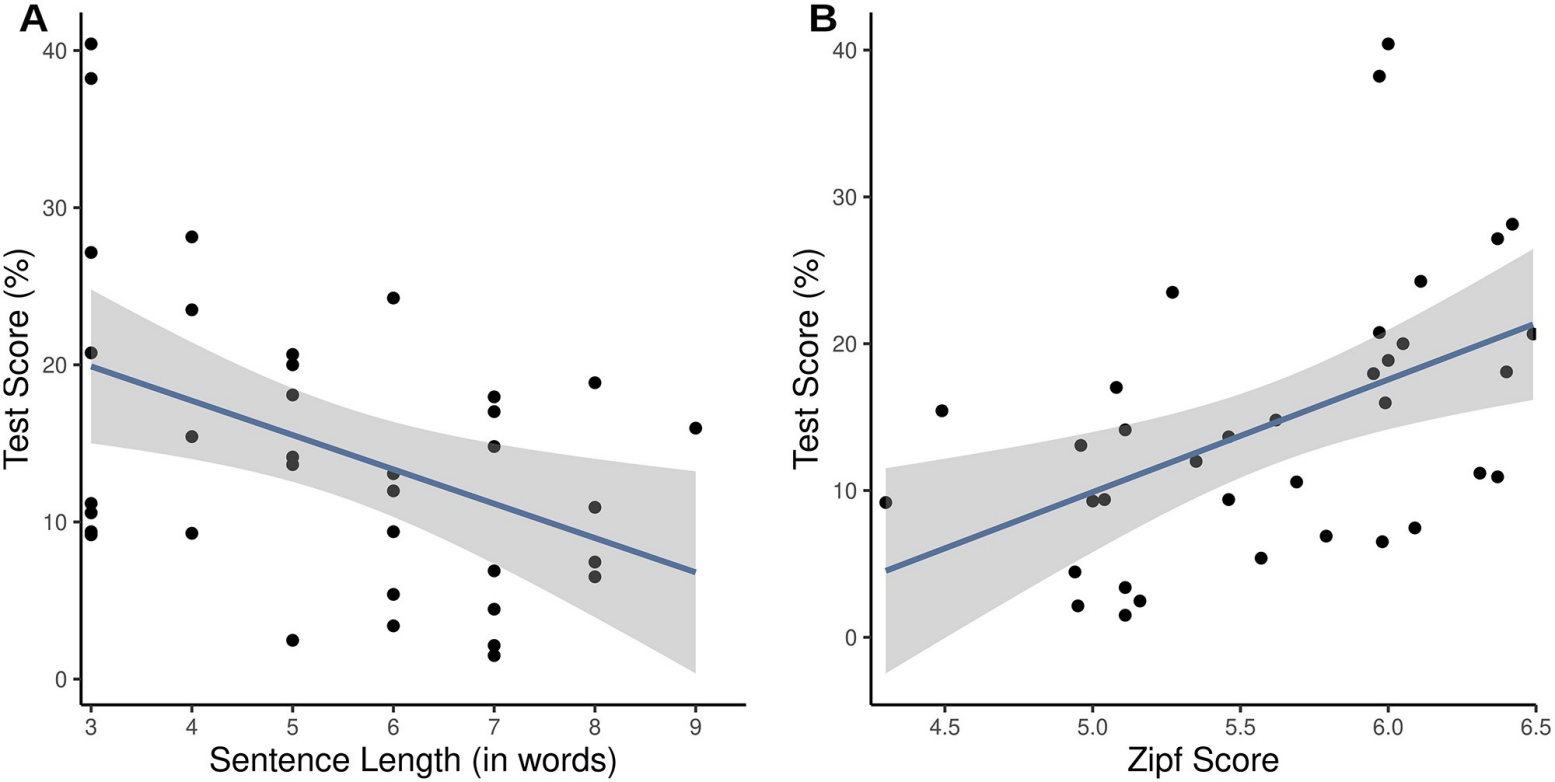
Impact of linguistic factors on the recognition score of sentences. A) Impact of sentence length on sentence recognition. Recognition scores decrease significantly with sentence length (p < .001). B) Impact of mean Zipf score on sentence recognition. The Zipf score of a sentence has a significant impact on the test score (p < .001). Higher Zipf scores predict higher recognition scores.

We then investigated if the main factors “sentence length” and “Zipf score” had an impact on the sentence recognition score and also if those main factors show an interaction. The mean score for short sentences was 20.27% (SD = 11.32%, range = 9.18–40.42%), for medium sentences 13.04% (SD = 6.99%, range = 2.47–24.25%) and for long sentences 10.37% (SD = 6.34%, range = 1.50–18.86%). There was a significant main effect of sentence length (*β* = -.164, SE = .002, *p <* .*001*), indicating that as sentence length increased, the word recognition score decreased (Fig 3A). We also found a significant main effect of the Zipf score (*β* = .590, SE = .006, *p <* .*001*), meaning that the mean sentence frequency can predict the recognition score of sentences (Fig 3B). We also found a significant interaction effect between the sentence length and the Zipf score (*β* = .05, SE = .003, *p <* .*001*), showing that the more words a sentence contains, the more the Zipf score has an influence on the recognition score. The table with the fixed effects can be found in the S7 Table.

### 3.2 Impact of hearing impairment

**Fig 4:**
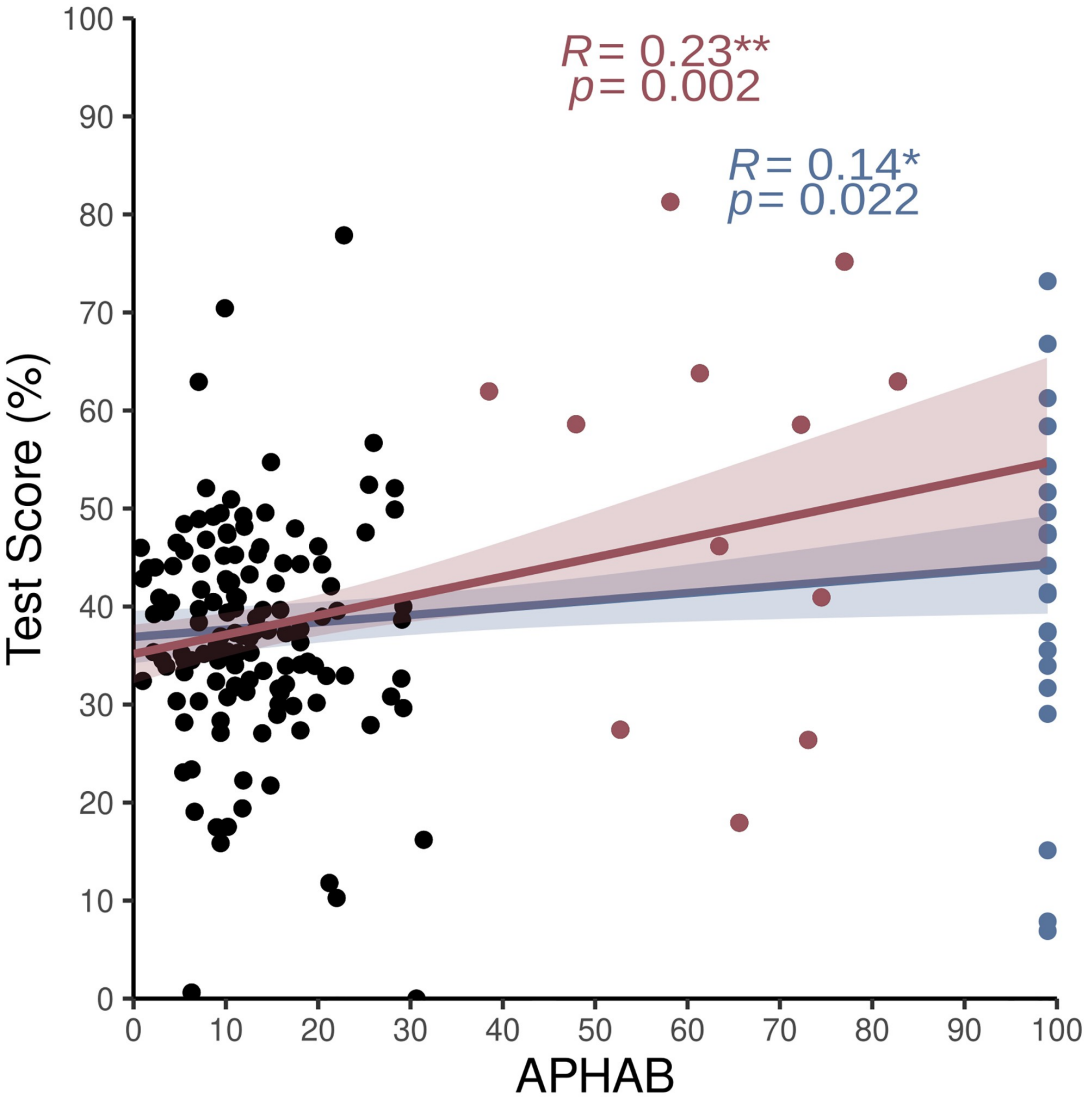
Influence of hearing impairment on the total score of each participant. Blue line indicates the relationship between self-reported hearing impairment and total score including prelingually deaf individuals (APHAB score = 99%, blue dots). Prelingual deaf individuals show much variation, but we still observe a positive relationship between hearing impairment and total score (η^2^ = .031, p = .022). Red line indicates the relationship between self-reported hearing impairment and total score excluding prelingually deaf individuals. Self-reported hearing impairment was low in general, but the sample also included people with more severe self-reported hearing impairment (red dots). We discovered a stronger relationship between self-reported hearing impairment and total score than for the whole sample (η^2^ = .061, p = .002).

Our previous analysis indicates that certain linguistic properties of the stimulus material influences lipreading performance. However, the interindividual variability is striking. In the next step, we describe how lipreading skills are related to hearing impairment. We started by calculating a regression model, in which subjective hearing impairment (APHAB) was the main factor and we tried to predict the influence on the total test score. We found a significant impact of the subjective hearing impairment on the total score (*β* = .195, SE = .063, *p =* .*002*, R^2^_adj_ = .05) in the sample with postlingual acquired hearing loss. The higher the participants rated their hearing impairment, the more they were able to recognize words by visual input alone (Fig 4). Assuming as a null hypothesis that prelingual deafness equals just an extreme version of postlingual hearing loss, we compared the predicted score (score at APHAB(99) = 54.46%) with the actual scores obtained by our prelingually deaf participants. In case the model based on the postlingually hearing sample is generalizable to the prelingually deaf group, the deviation from the predicted test score should be symmetrically clustered around zero. Contrary to this null hypothesis, we found that prelingually deaf subjects scored lower in the total lipreading score than expected from the sample with postlingual acquired hearing loss (*t*(21) = -10.04, *p = 1*.*81e-09*). We then decided to recalculate the model again for the whole sample. We found that including prelingually deaf people affects the relationship between hearing impairment and total test score (*β* = .075, SE = .033, *p =* .*02*, R^2^_adj_ = .03). Comparing the effect sizes of the models also revealed a stronger relationship between subjective hearing impairment and the total test score for the model just including acquired hearing loss (η^2^ = 0.06) than for the model including the whole sample (η^2^ = 0.03). A model on postlingual acquired hearing impairment therefore cannot account for prelingually acquired hearing loss, assuming basic differences between prelingual and postlingual hearing impairment.

To unravel if there are other factors influencing this relationship and how the groups differ, we calculated another regression model, again with the main factor subjective hearing impairment, and two moderator variables, namely age (since hearing loss increases with age) and education (assuming that high linguistic abilities contribute to better speech understanding) once for the group without prelingually deaf subjects and once for the whole sample. We did not find an impact of neither age (*β* = -.0008, SE = .004, *p =* .*85*) nor education (*β* = 0.02, SE = .04, *p =* .*52*) on the relationship between self-reported hearing impairment and total score in the group with postlingual acquired hearing loss. We also found no influence on the relationship for age (*β* = -.001, SE = .004, *p =* .*82*) in the whole sample. Descriptively a stronger influence on the relationship was observed for education, however, the effect was statistically not significant (*β* = .026, SE = .016, *p =* .*11*). Since education should be shown in the ability to report grammatically correct sentences and should therefore go in line with high literacy, we decided to calculate a new model with the same factors and moderator variables, but changing the dependent variable from “total test score” to “sentence score”. Here we found a significant influence of the moderator variable (*β* = .044, SE = .018, *p =* .*013*) on the relationship between hearing impairment and sentence score. A similar analysis using a linear mixed-effects model where all relevant variables are combined in one model can be found in the S8 Table.

### 3.3 Reduction of items and versions for SaLT

The current version of the test uses 18 numbers, 48 words and 36 sentences, which resulted in a test duration of about 30–50 minutes. Also we used 4 different versions that are randomly assigned to avoid that the effects are due to the speaker. We then decided to choose just 1 version and to minimize the number of items, in order to construct a more effective test (SaLT). For this, we fitted a Rasch model to our initial version of the test and used the itemfit and the Andersen LR-Test to eliminate items (see the section “Data analysis” for a more detailed description).

#### 3.3.1 Impact of version

We investigated the impact of the version on the total test score since the new release of SaLT is planned to include just one version of the original four versions used here. We calculated a Kruskal-Wallis test to test for differences between versions (Version 1: n = 46, Version 2: n = 34, Version 3: n = 42, Version 4: n = 48). We did not find significant differences (H(3) = 4.086, *p =* .*253*) between versions, suggesting no differences between the speakers as well. A similar analysis using a general linear mixed model and Post-Hoc Tukey contrasts can be found in the S9 and S10 Tables.

#### 3.3.2 Reduction of numbers

Using the stepwiseIt-function, we dropped 4 items as they showed significant deviation from the Rasch model (all *p <* .*005*). The remaining 14-item model revealed a satisfactory fit to the Rasch model. When testing for DIF, all items showed good outfit (all *t* < 1.21) and infit (all *t* < .96) with performance median as split criterion (all χ^2^(155) > 95.20, *p >* .*15*). Further testing of the itemfit based on the Andersen LR-test also revealed a satisfactory fit to the Rasch model (χ^2^(13) = 11.685, *p =* .*55*). A final analysis of internal consistency revealed an acceptable reliability of the items used (α = .75). Final itemfit statistics can be found in the S11 Table.

#### 3.3.3 Reduction of words

We separated our dataset of words into bilabial and non-bilabial words (each with 24 items) and fitted separate Rasch models to each set to make sure that the itemfit was not biased by the different item categories. In the bilabial set, we dropped 8 items using the stewiseIt-function as they showed significant deviation from the Rasch model (all *p <* .*04*). When testing for DIF, all items showed good outfit (all *t* < .30) and infit (all *t* < 1.12) with performance median as split criterion (all χ^2^(162) > 78.76, *p >* .*27*). Since we wanted to further minimize the number of items, we then tested the itemfit based on the Andersen LR-test, which revealed that 4 items were excluded by the function because of inappropriate response patterns within subgroups. After removing those items, the Andersen LR-test revealed a satisfactory fit of our 12-item model to the Rasch model (χ^2^(11) = 7.02, *p =* .*80*). Final itemfit statistics can be found in the S12 Table. In the non-bilabial set, we dropped 3 items using the stewiseIt-function as they showed significant deviation from the Rasch model (all *p <* .*01*). When testing for DIF, all items showed good outfit (all *t* < 1.28) and infit (all *t* < .93) with performance median as split criterion (all χ^2^(151) > 9.57, *p >* .*07*). Also here we wanted to further minimize the number of items, so we then tested the itemfit based on the Andersen LR-test, which revealed that 5 items were excluded by the function because of inappropriate response patterns within subgroups. After removing those items, the Andersen LR-test revealed a satisfactory fit of our 16-item model to the Rasch model (χ^2^(15) = 9.41, *p =* .*86*). Final itemfit statistics can be found in the S13 Table. A final analysis of internal consistency for all words (combining bilabial and non-bilabial words) revealed a high reliability of the items used (α = .80).

#### 3.3.4 Reduction of sentences

Performing an exploratory factor analysis for 1 factor (“lipreading abilities”) with a threshold of .50 for the factor loadings indicated that 14 items could be excluded because they did not display our latent trait. Therefore, our model consisted of 22 items that explained 31% of the variance with factor loadings from .50 to .78. A final analysis of internal consistency revealed an excellent reliability of the items used (α = .93). The table including all items and factor loadings can be found in the S14 Table. Items used in the new version are indicated in bold there.

## 4 Discussion

In the present study, we looked at linguistic factors and hearing impairment contributing to visual speech perception abilities. The recognition score between item categories (numbers, words and sentences) differed significantly. Numbers were recognized the most, followed by words and then by sentences. For words, the articulation (bilabial vs. non-bilabial) had an influence on the recognition score. While the frequency of a word used in the spoken language only has an influence if no bilabial cue (i.e. opening or closing of the mouth) is present, it has an influence on the sentence recognition score independent of the sentence length. The sentence length was also predictive of the recognition score, meaning that shorter sentences were recognized more than medium or long sentences. Also, longer sentences were recognized more often if they contained more frequently used words. Overall, we could not find a difference between different versions of the test with different speakers. Although our study shows high interpersonal variance in lipreading abilities in general, we did find an effect of hearing impairment on the total score, so the higher the self-rated hearing impairment was, the more items were recognized. Interestingly, this effect was even stronger when excluding deaf individuals, raising the question of how prelingual and postlingual hearing loss differentially impact lipreading skills. Moderator analyses with age and education unraveled an influence of education on the relationship between subjective hearing impairment and sentence score. Furthermore, we introduced a new German lipreading test which can be utilized to assess lipreading abilities in the general population, predominantly in studies that investigate visual speech perception.

### 4.1 Influential factors on item recognition

With a mean recognition rate of 68.43%, numbers were recognized the most, followed by words 33.62% and sentences 14.75%, showing a significant difference in mean recognition scores for the different item categories. The high score for numbers is plausibly due to the fact that providing participants with the context of “numbers to be recognized” reduces the number of possibilities for the solution (as they were told there were only 2-digit numbers). This goes in line with the hypothesis that lipreading abilities are higher when providing a certain context [14, 20, 40]), in our case a closed set of possible answers. A similar effect could be observed for words, although the number of recognized items was significantly lower than for numbers. Here we provide a wider set of possible answers, namely German monosyllabic words, which are used more frequently in spoken language than simple numbers, providing no reliable context information. Interestingly, the use-frequency of the words can only predict the recognition score when no visual cue (here the bilabial articulation) was presented. This also goes in line with a recent study stating that the opening and the closing of the mouth is a valuable cue for correctly identifying words [41]. This effect could build on the fact that labial phonemes are more visually salient and therefore easier to identify [16] But not only labial phonemes, but also labiodental consonants like /f/ and /w/ are very important cues in terms of visual speech perception [16]. Investigating how this consonant cluster differs from labial and non-labial consonants and controlling for the number of words with labiodental phonemes could have explained even more how participants use salient phonetic cues for advanced lipreading abilities, but this would have gone beyond the scope of the test construction. Another reason for the interaction between word frequency and articulation category could be that our approach did not take into account perceptual similarity [15, 42, 43] which could interfere with the frequency effect (words used more often in spoken language). Thus, people relied more on the movements of the mouth (bilabial vs. non-bilabial) in words and not on the use-frequency of the word, while in sentences, they relied on both the length and the average use-frequency of the sentence in spoken language. In our sentence stimuli, context was missing totally and they were also closest to a naturalistic setting where lipreading is needed, adding to the explanation of the low recognition score [14]. Another influential factor for those low scores in sentences could be the individual visual working memory span, as speechreading performance can be explained by scores in cognitive tasks [21], the size of the working memory and phonological processing abilities [14, 44, 45]. Our results go in line with the literature saying that the test score was related to sentence difficulty [46] as longer and less used sentences were recognized less often. Nevertheless, we find individual scores ranging from 0% to 75%, which could also strengthen the hypothesis by Summerfield [24] that “good speechreaders are born, not made”. Contradictory to this assumption, recent studies found that training and practice can enhance lipreading abilities in children, but decline without further training [47, 48] A recent invention of a speechreading test for deaf and normal hearing children [49] also highlighted that speechreading skills improved with age and there was no difference between normal hearing and hearing impaired children in terms of lipreading abilities, further supporting the notion that lipreading can indeed be learned. We did not find an influence of the speaker, since all 4 versions with differing speakers reached similar mean recognition scores, signifying that lipreading abilities are independent from the person whose lips are paid attention to. Also, when compared to a standardized synthetic talker, participants still have a higher recognition score for naturalistic stimuli from a human talker [16] This would suggest that natural differences in pronunciation occurring in human speakers may be neglectable.

### 4.2 Influence of hearing impairment

Investigating possible influences of subjective hearing impairment revealed that subjective hearing loss could predict lipreading abilities. The more hearing problems the participants reported, the higher was the total test score. Therefore, our results can strengthen the hypothesis of “perceptual compensation” [23] that states that higher hearing impairment results in a shift of attention from auditory speech cues to visual speech cues (since auditory cues are not as reliable as they used to be). People rely more on visual speech cues and as a consequence, they get better in visual speech perception, thus showing better lipreading abilities. Better lipreaders also have a higher success rate in rehabilitation after cochlear implantation [50], again pointing to the fact that hearing impairment triggers a perceptual compensation process important for optimal speech processing with diminished auditory input.

Assuming that prelingual hearing loss is a simple continuation of this (to a maximum increased hearing impairment) model and also in accordance with other observations of superior visual speech processing skills in the deaf population [19, 22, 51], prelingual deafness should be associated with enhanced lipreading skills. Applying a regression model trained on the postlingually hearing impaired individuals revealed that it does not generalize well to the prelingual group. Predicted performance was consistently lower than expected if prelingual deafness was seen as an equivalent of “extreme”postlingual hearing impairment. These results propose that the process of perceptual compensation seems to be absent or at least different in prelingually deaf people, resulting in different factors impacting lipreading abilities depending on the onset of deafness or hearing impairment. Studies introduce those factors as enhanced phonological processing [44] or verbal information processing skills [52].

Another study suggests that lipreading abilities correlate with reading abilities in both deaf and dyslexic populations [18] suggesting an impact of educational background. Our results go in line with this study by showing that the relationship between hearing impairment and sentence score is moderated by education. Interestingly, this interaction is absent when calculating the model just for the postlingually hearing impaired population, again pointing to the fact that prelingual and postlingual hearing loss is fundamentally different.

Thus, our findings show that especially in our group of prelingually deaf participants who do not use cochlear implants or hearing aids and rely mainly on sign language as a form of communication, education interacts with lipreading skills. Particular challenges could arise for this group in higher education where commonly oral language is the default, as sign language consists of grammatical structures other than spoken and written [53]. Therefore, on the one hand, lipreading skills might as a result be enhanced by the necessity of using oral language in higher educational settings. On the other hand, better lipreading skills might enable those individuals to stay longer on an educational pathway. Additionally, lipreading skills could also be linked to intelligence in prelingual deaf people [54], or can even moderate the relationship between education and lipreading abilities. Taken together, our findings could shed a light on why educational background interacts with liprading abilities in our sample of prelingually deaf people, but not in the sample with postlingual acquired hearing loss. It is again vital to mention that the sample of prelingually deaf participants tested here were exclusively chosen not to have received auditory input throughout their lifetime, a fact that may also impact the generalizability of our findings. How education influences congenitally deaf people with cochlear implants or hearing aids, needs to be discussed in further studies. Finally, it is noteworthy that despite our extensive analysis regarding the influential factors on enhanced lipreading abilities, we cannot fully explain the high variance in the assessment scored by prelingual deaf individuals.

Another important factor influencing lipreading abilities might be the duration of hearing impairment, as there is evidence that early-onset hearing impairment leads to better results when trying to understand visual speech [19] However, when analyzing the relationship between age and test score in our prelingual deaf (as age and duration of hearing impairment are identical), we did not find evidence for this notion in our group of prelingual deaf subjects (see also S1 Fig). But as this is only a small group of participants (N = 22), we cannot make general assumptions about the link between duration of hearing impairment and lipreading abilities. Furthermore, our results indicate that only little variance is accounted for by the self-reported hearing impairment, pointing to the fact that duration of hearing impairment could be a crucial parameter to further clarify how lipreading evolves over time depending on the severity of hearing loss and should be included in future studies.

We also have to consider the limitation of assessing hearing impairment within an online-study. Hearing impairment is usually measured by subjective (e.g. pure tone audiometry or speech audiometry, see Patterson and colleagues [55] and objective (e.g. auditory brainstem response, see Biacabe and colleagues [56]) audiometric investigations. Answering questions about everyday-life situations can thus present only a vast assumption of the actual hearing impairment participants are suffering from. Nevertheless, there has been evidence that people classify their hearing impairment at a rate of around 70% correct when comparing a subjective and objective hearing assessment [57].

While we cannot provide an objective measurement, we can still add evidence to a deeper understanding of how hearing loss and lipreading abilities are related in populations with variable subjective hearing problems.

### 4.3 SaLT: An openly available lipreading test

Aiming to provide an efficient visual speech perception assessment tool after our initial analysis on influential factors, we first decided on one version that will be used in the future. After comparing the 4 different versions used in the first release of the test, we decided to use the speakers from the version with the highest recognition score over all item categories (M = 40.65%, SD = 13.41) in the new SaLT 2.0. We then used the Rasch model for numbers and words and an EFA for sentences to remove non-fitting items. The final version of the Salzburg Lipreading Test includes 14 numbers, 28 monosyllabic words and 22 sentences and can be found on the OSF-page (see https://osf.io/sgj4n/). Thus, we reduced the total number of items from 102 to 64, resulting in a shortened version of the test by ~38%, providing a test which can be done online within 20 minutes and also comfortably prior to lab experiments (M/EEG, fMRI etc.). We still provide different items for the articulation category and frequency of words, and also for the different length and frequency categories of sentences, therefore still covering all investigated aspects that have an influence on the recognition score. Extended information on the items can be found in the supplementary material. Evaluating the internal consistency of our categories revealed a satisfactory internal reliability (Cronbach’s alpha for all categories > .80). Furthermore, we kept the APHAB questionnaire in the new version as it yields important insights into a possible hearing impairment condition from the participants (though it does not replace an objective measurement of hearing impairment). This screening tool can also be abandoned by the user if another appropriate hearing loss assessment is available.

## 5 Conclusion

Investigating the overall picture of this study revealed differential aspects contributing to visual word recognition for numbers, words and sentences. Different linguistic properties have different effects on simple word recognition or complex sentence recognition. While hearing impairment seems to alter lipreading abilities in the population being born with normal hearing, there seem to be other factors in the prelingual deaf population contributing to enhanced lipreading skills, in particular educational background. Further studies are needed to identify the aspects differentially affecting visual perception and the high variance in prelingual deaf and people with acquired postlingual hearing loss. The current study is also providing a new and reliable tool (SaLT) that can be used to assess visual speech perception abilities in the general population with an appropriate amount of items to be solved in as little time as possible.

## Supporting information

S1 Fig Relationship between age (= duration of hearing loss) and total test score for prelingually deaf individuals. Age and test score are not significantly correlated.
(DOCX)

S1 Table List of words presented to the participants. (DOCX)

S2 Table List of short sentences presented to the participants. (DOCX)

S3 Table List of medium long sentences presented to the participants. (DOCX)

S4 Table List of long sentences presented to the participants. (DOCX)

S5 Table Fixed effects table with recognition score as dependent variable. Signif. codes: 0 ’***’ 0.001 ’**’ 0.01 ’*’ 0.05 ’.’ 0.1 ’ ’ 1. *Note*: *Reference category for calculation was “Category*: *Numbers”*.
(DOCX)

S6 Table Fixed effects table with word recognition score as dependent variable. Signif. codes: 0 ’***’ 0.001 ’**’ 0.01 ’*’ 0.05 ’.’ 0.1 ’ ’ 1. *Note*: *Reference category for calculation was “Articulation category*: *Bilabial”*.
(DOCX)

S7 Table Fixed effects table with sentence recognition score as dependent variable. Signif. codes: 0 ’***’ 0.001 ’**’ 0.01 ’*’ 0.05 ’.’ 0.1 ’ ’ 1.
(DOCX)

S8 Table Fixed effects table with test score as dependent variable. Signif. codes: 0 ’***’ 0.001 ’**’ 0.01 ’*’ 0.05 ’.’ 0.1 ’ ’ 1. Note: Table shows the output from R (R Core Team, 2021).
(DOCX)

S9 Table Fixed effects table with recognition score as dependent variable. Signif. codes: 0 ’***’ 0.001 ’**’ 0.01 ’*’ 0.05 ’.’ 0.1 ’ ’ 1. Note: Reference category for calculation was “Version: Version 1”. Version 3 differs significantly from Version 1 in this calculation, but further calculating Tukey contrasts with Bonferroni correction revealed no significant differences.
(DOCX)

S10 Table Post-Hoc Tukey contrasts for differences between test versions. (DOCX)

S11 Table Itemfit statistics for numbers. Note: letter-number combinations are item-codes, and also the numbers presented to the participants in the test.
(DOCX)

S12 Table Itemfit statistics for bilabial words. Note: letter-number combinations are item-codes.
(DOCX)

S13 Table Itemfit statistics for non-bilabial words. Note: letter-number combinations are item-codes.
(DOCX)

S14 Table Exploratory factor analysis of the sentence items. Note: Extraction method: Minimal residual, Rotation method: Varimax. Loadings larger than .50 are in bold.
(DOCX)
