# Supplementary material for: Influence of linguistic properties and hearing impairment on visual speech perception skills in the German language
Source: PLoS One. 2022 Sep 30;17(9):e0275585. doi: 10.1371/journal.pone.0275585 (PMC9524625; doi:10.1371/journal.pone.0275585)
Supplement: S1 Fig — Age and test score are not significantly correlated. (DOCX) [file pone.0275585.s001.docx]

*
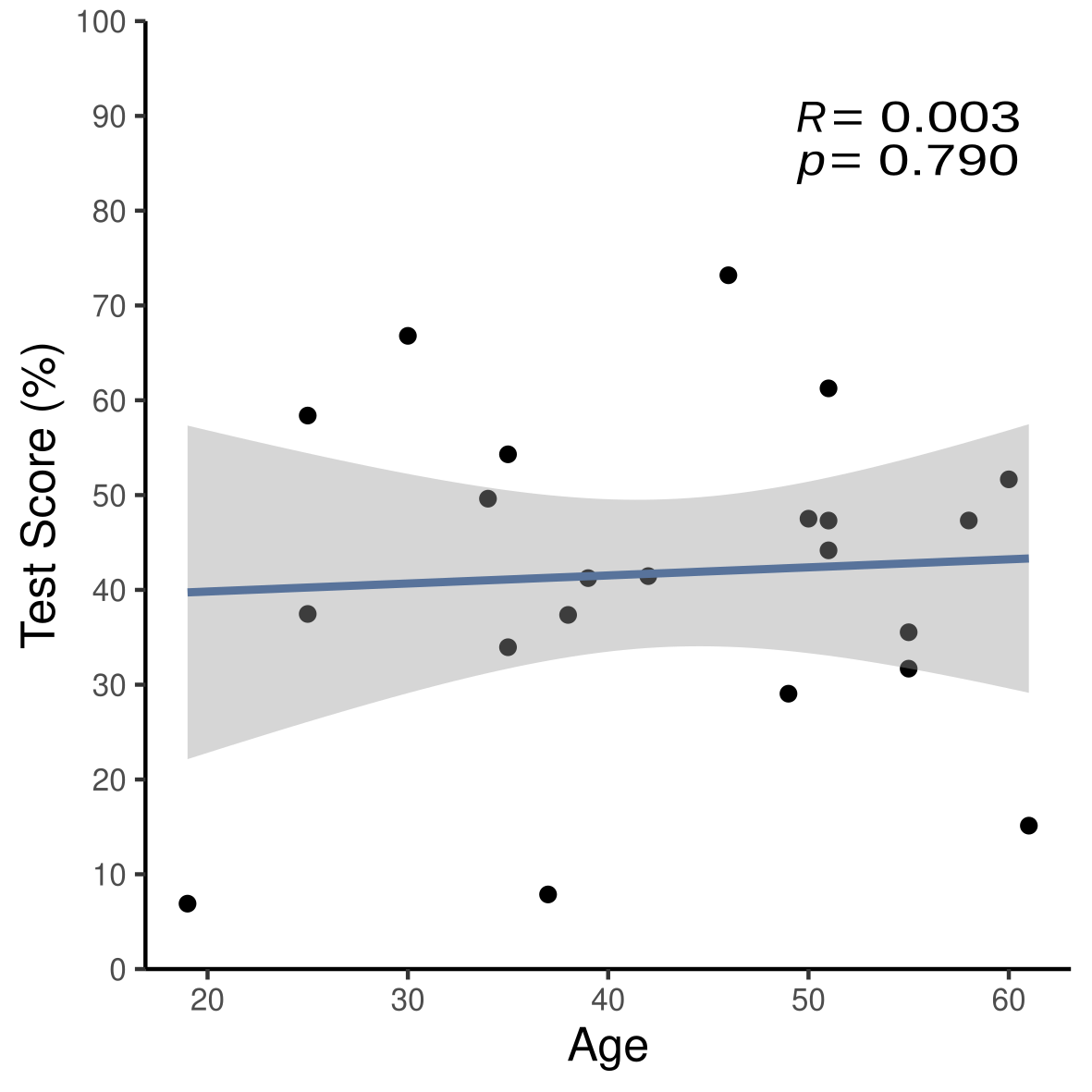
*

*Figure S1: Relationship between age (= duration of hearing loss) and total test score for prelingually deaf individuals. Age and test score are not significantly correlated.*
