# Supplementary material for: Influence of linguistic properties and hearing impairment on visual speech perception skills in the German language
Source: PLoS One. 2022 Sep 30;17(9):e0275585. doi: 10.1371/journal.pone.0275585 (PMC9524625; doi:10.1371/journal.pone.0275585)
Supplement: S1 Table — (DOCX) [file pone.0275585.s002.docx]

*Table S1: List of words presented to the participants*

| ***Bilabial*** | | | ***Non-bilabial*** | | |
| --- | --- | --- | --- | --- | --- |
| ***easy*** | ***medium*** | ***hard*** | ***easy*** | ***medium*** | ***hard*** |
| Mann | Blatt | Pflug | Uhr | Ei | Reif |
| Bild | Pferd | Moor | Herr | Herz | Klee |
| Bahn | Baum | Pfau | Zeit | Schatz | Schmutz |
| Bett | Berg | Pracht | Weg | Dorf | Dunst |
| Bier | Bauch | Pfahl | Frau | dumm | Hecht |
| Brot | Bank | Mahl | Fall | Licht | Gift |
| Band | Bach | Pflock | Gott | ernst | Thron |
| Mist | Pech | Molch | Geld | Gleis | Kies |
