# Supplementary material for: Influence of linguistic properties and hearing impairment on visual speech perception skills in the German language
Source: PLoS One. 2022 Sep 30;17(9):e0275585. doi: 10.1371/journal.pone.0275585 (PMC9524625; doi:10.1371/journal.pone.0275585)
Supplement: S2 Table — (DOCX) [file pone.0275585.s003.docx]

*Table S2: List of short sentences presented to the participants*

| ***Short sentences*** | | |
| --- | --- | --- |
| ***easy*** | ***medium*** | ***hard*** |
| Ich habe keine Ahnung. | Heute Nacht ist Vollmond. | Die Ampel ist ausgefallen. |
| Ist hier frei? | Verstehen Sie mich? | Wir wandern oft. |
| Mir ist schlecht. | Spielen Sie Karten? | Wann geschah der Unfall? |
| Ich komme später. | Haben Sie Schmerzen? | Beeil dich bitte. |
