# Supplementary material for: Influence of linguistic properties and hearing impairment on visual speech perception skills in the German language
Source: PLoS One. 2022 Sep 30;17(9):e0275585. doi: 10.1371/journal.pone.0275585 (PMC9524625; doi:10.1371/journal.pone.0275585)
Supplement: S3 Table — (DOCX) [file pone.0275585.s004.docx]

*Table S3: List of medium long sentences presented to the participants*

| ***Medium sentences*** | | |
| --- | --- | --- |
| ***easy*** | ***medium*** | ***hard*** |
| Es ist alles in Ordnung. | Mein Vater kann gut Geschichten erzählen. | Könntest du mir deine Jacke leihen? |
| Was macht ihr in den Ferien? | Möchtest du ein Museum besuchen? | Wurde der Brief gestern abgeschickt? |
| Sind Sie schon lange hier? | Wir könnten am Wochenende Freunde einladen. | Die Bäume verlieren nun ihre Blätter. |
| Das Buch ist sehr spannend. | Bleiben wir bei schlechtem Wetter hier? | Wann kommen endlich unsere Möbel? |
