# Supplementary material for: Influence of linguistic properties and hearing impairment on visual speech perception skills in the German language
Source: PLoS One. 2022 Sep 30;17(9):e0275585. doi: 10.1371/journal.pone.0275585 (PMC9524625; doi:10.1371/journal.pone.0275585)
Supplement: S4 Table — (DOCX) [file pone.0275585.s005.docx]

*Table S4: List of long sentences presented to the participants*

| ***Long sentences*** | | |
| --- | --- | --- |
| ***easy*** | ***medium*** | ***hard*** |
| Um wie viel Uhr sollen wir bei Ihnen sein? | Was kostet ein Doppelzimmer für eine Nacht? | Reichen Sie mir bitte ein Blatt Papier. |
| Ich rufe sie bestimmt später noch einmal an. | Hast du schon so einen Sonnenuntergang gesehen? | Der vergangene Sonntag war leider völlig verregnet. |
| Wenn ich Zeit habe, werde ich gerne kommen. | Im Stadion sind heute sehr viele Zuschauer. | An welchem Schalter kann man Postkarten erhalten? |
| Wie weit ist die Stadt von hier entfernt? | Ich hole Sie dann von Ihrem Hotel ab. | Auf dem Teich schwimmen viele kleine Enten. |
