# Supplementary material for: Influence of linguistic properties and hearing impairment on visual speech perception skills in the German language
Source: PLoS One. 2022 Sep 30;17(9):e0275585. doi: 10.1371/journal.pone.0275585 (PMC9524625; doi:10.1371/journal.pone.0275585)
Supplement: S5 Table — Signif. codes: 0 ’***’ 0.001 ’**’ 0.01 ’*’ 0.05 ’.’ 0.1 ’ ’ 1. Note: Reference category for calculation was “Category: Numbers”. (DOCX) [file pone.0275585.s006.docx]

*Table S5: Fixed effects table with recognition score as dependent variable*

| Predictor | Coef. *β* | SE (*β)* | ***z*** | ***p*** |
| --- | --- | --- | --- | --- |
| (Intercept) | 4.153 | .035 | 120.05 | < 2e-16 *** |
| Category: Words | -.711 | .016 | -43.72 | < 2e-16 *** |
| Category: Sentences | -1.535 | .022 | -69.53 | < 2e-16 *** |

Signif. codes: 0 '***' 0.001 '**' 0.01 '*' 0.05 '.' 0.1 ' ' 1

*Note: Reference category for calculation was “Category: Numbers”.*
