# Supplementary material for: Influence of linguistic properties and hearing impairment on visual speech perception skills in the German language
Source: PLoS One. 2022 Sep 30;17(9):e0275585. doi: 10.1371/journal.pone.0275585 (PMC9524625; doi:10.1371/journal.pone.0275585)
Supplement: S6 Table — Signif. codes: 0 ’***’ 0.001 ’**’ 0.01 ’*’ 0.05 ’.’ 0.1 ’ ’ 1. Note: Reference category for calculation was “Articulation category: Bilabial”. (DOCX) [file pone.0275585.s007.docx]

*Table S6: Fixed effects table with word recognition score as dependent variable*

| Predictor | Coef. *β* | SE (*β)* | ***z*** | ***p*** |
| --- | --- | --- | --- | --- |
| (Intercept) | 3.567 | .054 | 66.53 | < 2e-16 *** |
| Zipf score | -.034 | .002 | -14.26 | < 2e-16 *** |
| Articulation category: Other | -.463 | .004 | -113.52 | < 2e-16 *** |
| Zipf score * Articulation category: Other | .226 | .003 | 62.28 | < 2e-16 *** |

Signif. codes: 0 '***' 0.001 '**' 0.01 '*' 0.05 '.' 0.1 ' ' 1

*Note: Reference category for calculation was “Articulation category: Bilabial”.*
