# Supplementary material for: Influence of linguistic properties and hearing impairment on visual speech perception skills in the German language
Source: PLoS One. 2022 Sep 30;17(9):e0275585. doi: 10.1371/journal.pone.0275585 (PMC9524625; doi:10.1371/journal.pone.0275585)
Supplement: S7 Table — Signif. codes: 0 ’***’ 0.001 ’**’ 0.01 ’*’ 0.05 ’.’ 0.1 ’ ’ 1. (DOCX) [file pone.0275585.s008.docx]

*Table S7: Fixed effects table with sentence recognition score as dependent variable*

| Predictor | Coef. *β* | SE (*β)* | ***z*** | ***p*** |
| --- | --- | --- | --- | --- |
| (Intercept) | 1.827 | .136 | 13.38 | < 2e-16 *** |
| Zipf score | .590 | .006 | 85.36 | < 2e-16 *** |
| Sentence length | -.164 | .002 | -77.77 | < 2e-16 *** |
| Zipf score * Sentence length | .058 | .003 | 14.58 | < 2e-16 *** |

Signif. codes: 0 '***' 0.001 '**' 0.01 '*' 0.05 '.' 0.1 ' ' 1
