# Supplementary material for: Influence of linguistic properties and hearing impairment on visual speech perception skills in the German language
Source: PLoS One. 2022 Sep 30;17(9):e0275585. doi: 10.1371/journal.pone.0275585 (PMC9524625; doi:10.1371/journal.pone.0275585)
Supplement: S8 Table — Signif. codes: 0 ’***’ 0.001 ’**’ 0.01 ’*’ 0.05 ’.’ 0.1 ’ ’ 1. Note: Table shows the output from R (R Core Team, 2021). (DOCX) [file pone.0275585.s009.docx]

*Table S8: Fixed effects table with test score as dependent variable*

| Predictor | Estimate | Std. Error | df | t-value | Pr(>\|t\|) |
| --- | --- | --- | --- | --- | --- |
| (Intercept) | 9.27680 | 2.90311 | 168.45397 | 3.195 | 0.001667 ** |
| Item category | 23.69062 | 0.64406 | 161.60929 | 36.783 | < 2e-16 *** |
| APHAB | 0.15670 | 0.04176 | 192.22375 | 3.753 | 0.000232 *** |
| Age | 0.18469 | 0.07763 | 164.59408 | 2.379 | 0.018499 * |
| Education | 1.28125 | 0.59538 | 190.14874 | 2.152 | 0.032658 * |
| APHAB * Education | 0.04430 | 0.01682 | 192.96496 | 2.634 | 0.009132 ** |
| Item category * APHAB | -0.04199 | 0.02384 | 161.17224 | -1.761 | 0.080059 . |
| Item category * Education | 0.45011 | 0.33182 | 161.84019 | 1.357 | 0.176829 |
| Item category * APHAB * Education | -0.01808 | 0.00972 | 161.15377 | -1.860 | 0.064652 . |

Signif. codes: 0 '***' 0.001 '**' 0.01 '*' 0.05 '.' 0.1 ' ' 1

*Note: Table shows the output from R (R Core Team, 2021)*
