# Supplementary material for: Influence of linguistic properties and hearing impairment on visual speech perception skills in the German language
Source: PLoS One. 2022 Sep 30;17(9):e0275585. doi: 10.1371/journal.pone.0275585 (PMC9524625; doi:10.1371/journal.pone.0275585)
Supplement: S9 Table — Signif. codes: 0 ’***’ 0.001 ’**’ 0.01 ’*’ 0.05 ’.’ 0.1 ’ ’ 1. Note: Reference category for calculation was “Version: Version 1”. Version 3 differs significantly from Version 1 in this calculation, but further calculating Tukey contrasts with Bonferroni correction revealed no significant differences. (DOCX) [file pone.0275585.s010.docx]

*Table S9: Fixed effects table with recognition score as dependent variable*

| Predictor | Coef. *β* | SE (*β)* | ***z*** | ***p*** |
| --- | --- | --- | --- | --- |
| (Intercept) | 3.650 | .068 | 53.570 | < 2e-16 *** |
| Version: Version 2 | -.057 | .104 | -.545 | .586 |
| Version: Version 3 | -.213 | .099 | -2.162 | .031 * |
| Version: Version 4 | -.006 | .094 | -.066 | .947 |

Signif. codes: 0 '***' 0.001 '**' 0.01 '*' 0.05 '.' 0.1 ' ' 1

*Note: Reference category for calculation was “Version: Version 1”. Version 3 differs significantly from Version 1 in this calculation, but further calculating Tukey contrasts with Bonferroni correction revealed no significant differences.*
