# Supplementary material for: Influence of linguistic properties and hearing impairment on visual speech perception skills in the German language
Source: PLoS One. 2022 Sep 30;17(9):e0275585. doi: 10.1371/journal.pone.0275585 (PMC9524625; doi:10.1371/journal.pone.0275585)
Supplement: S10 Table — (DOCX) [file pone.0275585.s011.docx]

*Table S10: Post-Hoc Tukey contrasts for differences between test versions*

| **Contrast** | **Estimate** | **SE** | **z** | **p_Bonferroni_** |
| --- | --- | --- | --- | --- |
| V2 - V1 | -.057 | .104 | -0.545 | 1.000 |
| V3 - V1 | -.213 | .099 | -2.162 | .184 |
| V4 - V1 | -.006 | .094 | -.066 | 1.000 |
| V3 - V2 | -.156 | .106 | -1.472 | .845 |
| V4 - V2 | .050 | .102 | ´.494 | 1.000 |
| V4 - V3 | .207 | .097 | 2.142 | .193 |
