# Supplementary material for: Influence of linguistic properties and hearing impairment on visual speech perception skills in the German language
Source: PLoS One. 2022 Sep 30;17(9):e0275585. doi: 10.1371/journal.pone.0275585 (PMC9524625; doi:10.1371/journal.pone.0275585)
Supplement: S11 Table — Note: letter-number combinations are item-codes, and also the numbers presented to the participants in the test. (DOCX) [file pone.0275585.s012.docx]

*Table S11: Itemfit statistics for numbers*

|  | **Chisq** | **df** | **p-value** | **Outfit MSQ** | **Infit MSQ** | **Outfit t** | **Infit t** | **Discrim** |
| --- | --- | --- | --- | --- | --- | --- | --- | --- |
| **z22** | 127.855 | 155 | 0.946 | 0.820 | 0.963 | -0.717 | -0.229 | 0.348 |
| **z33** | 129.952 | 155 | 0.929 | 0.833 | 0.905 | -0.416 | -0.577 | 0.213 |
| **z43** | 141.969 | 155 | 0.765 | 0.910 | 0.976 | -0.637 | -0.318 | 0.348 |
| **z45** | 138.060 | 155 | 0.832 | 0.885 | 0.906 | -0.785 | -1.097 | 0.479 |
| **z46** | 122.694 | 155 | 0.974 | 0.787 | 0.89 | -0.968 | -0.852 | 0.451 |
| **z47** | 95.198 | 155 | 1.000 | 0.610 | 0.737 | -1.460 | -1.728 | 0.537 |
| **z51** | 173.061 | 155 | 0.152 | 1.109 | 1.055 | 0.878 | 0.744 | 0.271 |
| **z54** | 114.741 | 155 | 0.993 | 0.736 | 0.827 | -0.786 | -0.973 | 0.452 |
| **z63** | 123.527 | 155 | 0.970 | 0.792 | 0.866 | -1.363 | -1.711 | 0.413 |
| **z71** | 170.157 | 155 | 0.192 | 1.091 | 1.052 | 0.773 | 0.736 | 0.282 |
| **z80** | 151.284 | 155 | 0.569 | 0.970 | 1.052 | -0.098 | 0.584 | 0.195 |
| **z86** | 161.176 | 155 | 0.351 | 1.033 | 1.014 | 0.304 | 0.227 | 0.279 |
| **z98** | 148.447 | 155 | 0.633 | 0.952 | 0.999 | -0.401 | 0.012 | 0.323 |
| **z99** | 178.171 | 155 | 0.098 | 1.142 | 1.062 | 1.216 | 0.962 | 0.238 |

*Note: letter-number combinations are item-codes, and also the numbers presented to the participants in the test*
