# Supplementary material for: Influence of linguistic properties and hearing impairment on visual speech perception skills in the German language
Source: PLoS One. 2022 Sep 30;17(9):e0275585. doi: 10.1371/journal.pone.0275585 (PMC9524625; doi:10.1371/journal.pone.0275585)
Supplement: S12 Table — Note: letter-number combinations are item-codes. (DOCX) [file pone.0275585.s013.docx]

*Table S12: Itemfit statistics for bilabial words*

|  | **Chisq** | **df** | **p-value** | **Outfit MSQ** | **Infit MSQ** | **Outfit t** | **Infit t** | **Discrim** |
| --- | --- | --- | --- | --- | --- | --- | --- | --- |
| **bl11** | 153.346 | 144 | 0.282 | 1.058 | 1.001 | 0.390 | 0.047 | 0.241 |
| **bl33** | 132.017 | 144 | 0.754 | 0.910 | 0.965 | -0.866 | -0.464 | 0.365 |
| **bl47** | 143.469 | 144 | 0.497 | 0.989 | 0.922 | -0.048 | -0.999 | 0.455 |
| **bl8** | 94.771 | 144 | 0.999 | 0.654 | 0.927 | -0.826 | -0.247 | 0.260 |
| **bm69t** | 168.75 | 144 | 0.078 | 1.164 | 1.107 | 0.996 | 1.076 | 0.095 |
| **bm81** | 133.809 | 144 | 0.718 | 0.923 | 0.953 | -0.772 | -0.65 | 0.327 |
| **bm8k** | 169.155 | 144 | 0.075 | 1.167 | 0.897 | 0.508 | -0.357 | 0.221 |
| **bm87** | 114.676 | 144 | 0.966 | 0.791 | 0.915 | -0.976 | -0.663 | 0.386 |
| **bm89** | 114.515 | 144 | 0.967 | 0.79 | 0.820 | -1.180 | -1.748 | 0.519 |
| **bm96** | 124.187 | 144 | 0.882 | 0.856 | 0.970 | -0.651 | -0.209 | 0.348 |
| **bs339** | 154.386 | 144 | 0.262 | 1.065 | 1.08 | 0.674 | 1.132 | 0.170 |
| **bs383l** | 154.42 | 144 | 0.261 | 1.065 | 1.059 | 0.638 | 0.818 | 0.156 |

*Note: letter-number combinations are item-codes*
