# Supplementary material for: Influence of linguistic properties and hearing impairment on visual speech perception skills in the German language
Source: PLoS One. 2022 Sep 30;17(9):e0275585. doi: 10.1371/journal.pone.0275585 (PMC9524625; doi:10.1371/journal.pone.0275585)
Supplement: S13 Table — Note: letter-number combinations are item-codes. (DOCX) [file pone.0275585.s014.docx]

*Table S13: Itemfit statistics for non-bilabial words*

|  | **Chisq** | **df** | **p-value** | **Outfit MSQ** | **Infit MSQ** | **Outfit t** | **Infit t** | **Discrim** |
| --- | --- | --- | --- | --- | --- | --- | --- | --- |
| **nl12** | 139.22 | 151 | 0.745 | 0.916 | 0.955 | -0.827 | -0.676 | 0.317 |
| **nl4** | 94.144 | 151 | 1.000 | 0.619 | 0.822 | -0.956 | -0.765 | 0.411 |
| **nl5** | 133.49 | 151 | 0.844 | 0.878 | 0.949 | -1.001 | -0.705 | 0.343 |
| **nl6** | 143.561 | 151 | 0.654 | 0.944 | 0.978 | -0.521 | -0.318 | 0.255 |
| **nl7** | 138.178 | 151 | 0.765 | 0.909 | 0.949 | -0.200 | -0.238 | 0.319 |
| **nl9** | 172.297 | 151 | 0.113 | 1.134 | 0.944 | 0.651 | -0.390 | 0.369 |
| **nm68** | 126.889 | 151 | 0.924 | 0.835 | 0.927 | -0.895 | -0.629 | 0.389 |
| **nm70** | 115.923 | 151 | 0.985 | 0.763 | 0.948 | -0.418 | -0.128 | 0.244 |
| **nm71** | 172.738 | 151 | 0.109 | 1.136 | 1.075 | 1.278 | 1.064 | 0.199 |
| **nm75** | 146.294 | 151 | 0.593 | 0.962 | 1.084 | -0.061 | 0.596 | 0.183 |
| **nm77** | 138.737 | 151 | 0.754 | 0.913 | 0.886 | 0.095 | -0.200 | 0.236 |
| **ns333** | 114.82 | 151 | 0.987 | 0.755 | 0.936 | -0.986 | -0.397 | 0.344 |
| **ns334** | 143.873 | 151 | 0.647 | 0.947 | 0.921 | -0.050 | -0.363 | 0.317 |
| **ns335** | 148.017 | 151 | 0.553 | 0.974 | 0.957 | -0.227 | -0.625 | 0.333 |
| **ns337** | 152.393 | 151 | 0.453 | 1.003 | 1.019 | 0.178 | 0.161 | 0.086 |
| **ns344** | 88.516 | 151 | 1.000 | 0.582 | 0.741 | -0.600 | -0.744 | 0.431 |

*Note: letter-number combinations are item-codes*
