# Supplementary material for: Influence of linguistic properties and hearing impairment on visual speech perception skills in the German language
Source: PLoS One. 2022 Sep 30;17(9):e0275585. doi: 10.1371/journal.pone.0275585 (PMC9524625; doi:10.1371/journal.pone.0275585)
Supplement: S14 Table — Note: Extraction method: Minimal residual, Rotation method: Varimax. Loadings larger than .50 are in bold. (DOCX) [file pone.0275585.s015.docx]

*Table S14: Exploratory factor analysis of the sentence items*

| ***Items*** | ***Factor*** |
| --- | --- |
|  | ***1*** |
| kl376 | **0.689** |
| kl52 | 0.190 |
| kl63 | **0.609** |
| kl73 | 0.464 |
| km127 | 0.499 |
| km224 | **0.541** |
| km234 | **0.590** |
| km413 | **0.557** |
| ks119 | **0.533** |
| ks195 | **0.500** |
| ks263 | 0.375 |
| ks32 | 0.360 |
| ll352 | **0.612** |
| ll427 | **0.735** |
| ll437 | **0.603** |
| ll485 | **0.556** |
| lm135 | **0.699** |
| lm229 | **0.556** |
| lm29 | **0.549** |
| lm505 | 0.476 |
| ls134 | 0.332 |
| ls166 | 0.497 |
| ls286 | 0.474 |
| ls507 | 0.475 |
| ml117 | **0.576** |
| ml264 | **0.599** |
| ml38 | **0.582** |
| ml69 | 0.493 |
| mm107 | 0.258 |
| mm114 | **0.714** |
| mm49 | **0.781** |
| mm94 | **0.743** |
| ms104 | **0.683** |
| ms359 | 0.496 |
| ms36 | 0.418 |
| ms572 | **0.579** |

*Note: Extraction method: Minimal residual, Rotation method: Varimax. Loadings larger than .50 are in bold*
